# Ultrasound-guided versus conventional sinus tract laser closure for adolescent sacrococcygeal pilonidal disease: a retrospective comparative study

**DOI:** 10.1007/s10151-026-03398-2

**Published:** 2026-07-30

**Authors:** Hongwei Hua, Zhicheng Li, Lei Jin, Mengqing Dong, Qinghan Ma, Xingli Xu, Jialin Qin, Zhenyi Wang, Jiong Wu

**Affiliations:** Department of Proctology, Yueyang Hospital of Integrated Traditional Chinese and Western Medicine, Shanghai University of Traditional Chinese Medicine, Shanghai, 200437 China

**Keywords:** Pilonidal sinus, Laser therapy, Ultrasound guidance, Adolescents, Recurrence

## Abstract

**Background:**

Sacrococcygeal pilonidal disease (SPD) is common in adolescents and often recurs after surgery. Sinus tract laser closure (SiLaC) helps with healing, reduces recurrence, and improves appearance but may miss sinus branches. Using ultrasound guidance during SiLaC ensures precise treatment and protects healthy tissue. This study compared the short- and medium-term efficacy of ultrasound (US)-guided SiLaC with conventional SiLaC in treating adolescent patients with SPD.

**Methods:**

This retrospective study gathered and analyzed clinical data from 64 adolescent (18 years or younger) patients with SPD who underwent either SiLaC or US-guided SiLaC at a single center from March 2021 to January 2025. The study included 38 patients who received conventional SiLaC and 26 patients who received US-guided SiLaC, and recorded demographics, surgical history, lifestyle factors, laser parameters, short-term complications, healing and recurrence rates, wound closure time, and recovery time.

**Results:**

Among the 64 patients, 89.1% were male and 10.9% female, with a median age of 17 years. The US-guided group had a median follow-up of 18.0 (8.0, 37.8) months, while the conventional group had 20.5 (9.5, 35.8) months, both groups with similar demographic characteristics. The US-guided group showed significantly lower intraoperative laser duration and energy use (*p* < 0.05), and faster wound closure (*p* = 0.017). Recurrence was 4% in the US group vs. 25% in the conventional group (*p* = 0.037). No significant difference was found in the time to resume normal activities (*p* = 0.091).

**Conclusions:**

Preliminary results suggest that US-guided SiLaC enhances the precision of sinus tract ablation by providing real-time intraoperative visualization in adolescents with SPD. In this cohort, its application was associated with reduced recurrence rates and expedited wound healing. However, these findings necessitate validation through larger-scale studies.

## Introduction

Sacrococcygeal pilonidal disease (SPD) predominantly affects young male individuals, with a male-to-female ratio of 2–4:1 and typical onset between ages 14 and 25 [1–4]. Its incidence has risen over recent decades to approximately 40 per 100,000 person-years [3]. SPD involves midline pits in the gluteal cleft caused by hair penetrating the skin. In the acute phase, patients experience rapid erythema and pain, sometimes with discharge or fever [4, 5]. The chronic phase features recurrent abscesses and discharge over months or years, significantly affecting quality of life and potentially causing psychological distress [6]. Adolescent patients experience a disproportionately high disease burden, characterized by an increasing incidence and notably earlier and higher postoperative recurrence rates compared to adult populations [7]. This condition adversely affects growth, development, and academic performance as a result of persistent symptoms and the intricacies of postoperative care [8]. Given the necessity for adolescents to achieve rapid recovery, minimal trauma, optimal cosmetic outcomes, and low recurrence rates to prevent repeated interventions, there is an urgent need for minimally invasive and precise treatment strategies.

Although multiple treatment options are available, no single approach has been universally accepted as the optimal treatment for all presentations of SPD [1, 4]. Asymptomatic SPD does not need surgery, but symptomatic cases often require it. Acute abscesses need prompt drainage, while stable cases are treated with primary closure, open healing, or flap techniques [9]. Primary closure speeds up recovery but has higher recurrence, whereas secondary intention reduces recurrence but requires extended wound care [10–12]. Flap procedures such as Karydakis, Limberg, Z-plasty, and Bascom cleft lift generally have low recurrence rates and better cosmetic outcomes [1, 10]. The Bascom cleft lift is notably efficient, with a short surgery time and positive long-term results [13]. For adolescents, where quick recovery and minimal tissue trauma are crucial to prevent disruption of school and activities, minimally invasive techniques are being developed to meet these needs [14].

At present, minimally invasive interventions for SPD dominantly encompass endoscopic pilonidal sinus treatment (EPSiT), video-assisted ablation of pilonidal sinus (VAAPS), and laser ablation techniques [15–17]. Among these, sinus tract laser closure (SiLaC) is a notable minimally invasive surgical method for laser treatment of SPD [17]. Dessily et al. first applied SiLaC for SPD in 2014, reporting a success rate of 87.5% in 40 patients [18]. Subsequent studies have shown healing rates of 91.7% to 96.7% for SiLaC in SPD [17, 19–21]. Our center has extensive experience with laser ablation for SPD, having performed over 1000 procedures. A previous study involving nearly 400 patients from our center showed a wound healing rate of 100% and a recurrence rate of only 5.1% [22]. Although SiLaC for SPD is characterized by high success rates, few postoperative complications, and easy wound management, the inability to directly visualize the sinus tract during the procedure may result in incomplete laser ablation or missed side branches, potentially leading to recurrence [23, 24]. In particular, our preliminary clinical observations of SiLaC have identified age ≤ 18 years as an independent risk factor for postoperative recurrence in male patients [22]. This observation has led us to initiate advancements in the SiLaC technique tailored specifically for the adolescent population.

Ultrasound (US) is an important modality for diagnosing SPD; preoperative ultrasound accurately identifies the extent, shape, and branches of the pilonidal sinus, helping surgeons precisely locate the surgical site [25]. Intraoperative ultrasound plays a crucial role in the real-time visualization of the entire sinus tract and its side branches during ablation procedures, facilitating the complete eradication of lesions and minimizing the risk of residual diseased tissue. Papagiannopoulos et al. pioneered the integration of ultrasound guidance in laser treatment for SPD, termed the US-guided Si.La.D technique, and demonstrated its efficacy and safety in a limited cohort of patients without age-specific analysis [24]. However, no extensive or systematic studies have been conducted to compare the efficacy, safety, wound healing, and recurrence rates of US-guided SiLaC versus conventional SiLaC specifically in adolescent populations with SPD.

Given the high recurrence risk and distinct clinical needs of adolescents with SPD, this study employed SiLaC with adjunctive ultrasound guidance and compared it to SiLaC alone for treating SPD. The primary objectives were to evaluate and compare recurrence rates, wound closure times, and intraoperative laser parameters between the two treatment groups. The aim was to provide evidence-based insights into precision minimally invasive treatment for adolescent pilonidal disease and to generate robust preliminary data for the design of future large-scale prospective clinical trials.

## Materials and methods

This retrospective comparative cohort study included consecutive adolescent patients with SPD who underwent either conventional SiLaC (conventional group) or US-guided SiLaC (ultrasound group) at our institution between March 2021 and January 2025. Data were extracted from electronic medical records. Our center initiated the use of laser therapy for SPD in 2019. Follow-up of initial cases revealed a high rate of postoperative recurrence in the adolescent population, which we hypothesized was attributable to the incomplete ablation of minor sinus tracts. Consequently, in early 2021, we incorporated intraoperative ultrasound guidance to augment the laser ablation procedure. Preoperatively, both surgical modalities were thoroughly explained to the patients and their guardians, with the final treatment allocation determined by their informed choice.

Inclusion criteria were (1) age ≤ 18 years, any sex; (2) preoperative diagnosis of SPD confirmed by physical examination and ultrasonography or magnetic resonance imaging (MRI); (3) underwent either SiLaC or US-guided SiLaC.

The exclusion criterion was an acute abscess phase.

**Fig. 1:**
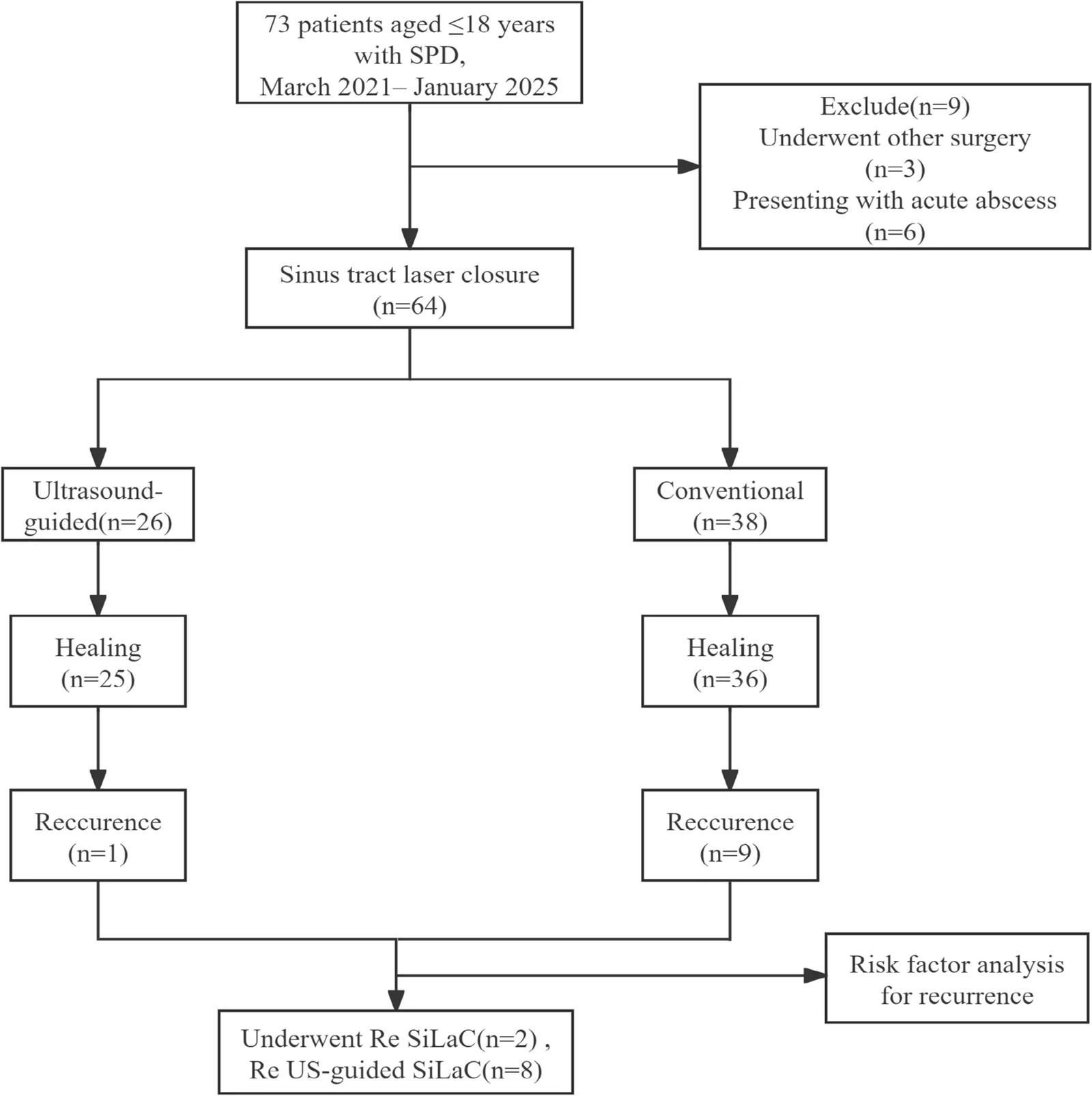
Study flow diagram

Before treatment, the surgical procedure, potential risks, and possible complications were thoroughly explained to the patients and their families. Written informed consent was obtained from the legal guardians of all patients after a detailed explanation of the surgical technique and associated risks. This study adhered to the principles of the Declaration of Helsinki and was approved by the Ethics Committee of Yueyang Hospital of Integrated Traditional Chinese and Western Medicine, Shanghai University of Traditional Chinese Medicine (Approval No. 2017-033). Figure 1 presents the study flow diagram.

### Techniques

#### Conventional group (sinus tract laser closure)

All patients underwent preoperative shaving of the sacrococcygeal region. The surgical procedure adhered to a standardized protocol as follows: Patients were positioned laterally, and intravenous anesthesia was administered via target-controlled infusion. Induction was achieved using a propofol medium- and long-chain triglyceride emulsion at a concentration of 3 μg/ml, individualized on the basis of the patient’s age, height, and weight, with maintenance at 2 μg/ml. The exploration commenced at the midline pit closest to the anal verge, utilizing a probe to ascertain the direction and course of the sinus tract. In cases where multiple pits were present, each was explored individually to assess their anatomical relationships. An incision, either circular or elliptical, was made at the apex of the sinus tract, adjusted according to the extent of infection. Mosquito forceps or a curette were employed through the incision to excise hair and necrotic tissue from the sinus tract. Subsequently, an optical fiber catheter (Leonardo Dual 45 Laser, CeramOptec GmbH of Biolitec AG, Bonn, Germany, single radial laser fiber) was introduced through the pit into the single-ring laser catheter. Under guidance from the aiming beam, the fiber was advanced to the end of the sinus tract (the incision site). The sinus tract was ablated and closed by withdrawing the catheter at a constant speed of 1 mm/s using a wavelength of 1470 nm and power of 10 W.

#### Ultrasound group (ultrasound-guided sinus tract laser closure)

**Fig. 2:**
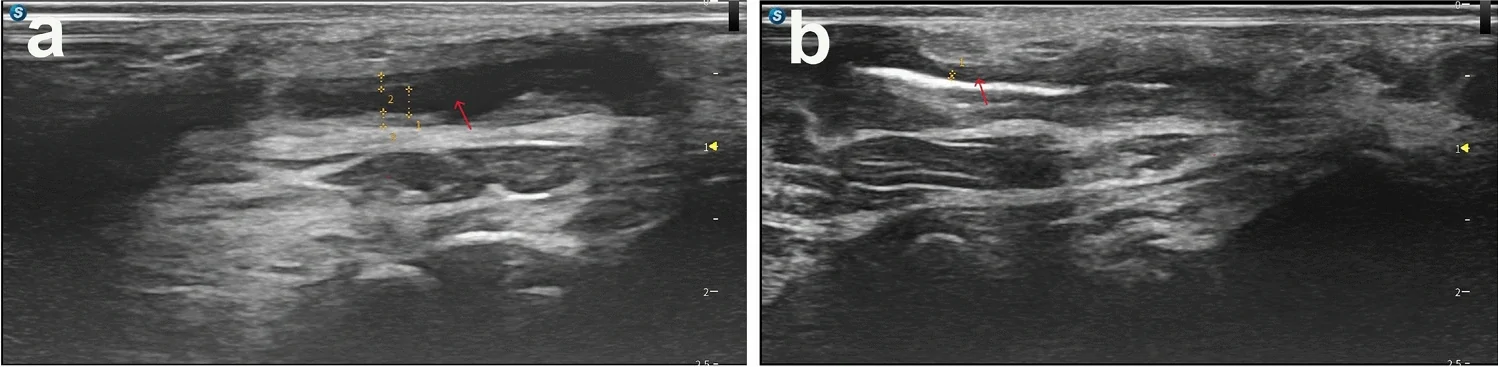
Perioperative ultrasound images of SPD during the stable phase in a 16-year-old male patient. Red arrows indicate the location of the sinus tract. **a** Immediately preoperative. **b** Immediately postoperative

The anesthesia procedure was the same as for SiLaC. The surgical procedure was essentially the same as that for the conventional group, with the addition of ultrasound guidance as follows:

Under real-time guidance using a 12-MHz linear ultrasound probe (X11 Portable Doppler Ultrasound Diagnostic System, SonoScape Medical Corp., Shenzhen, China), the sinus tract direction, diameter, length, and extent were explored starting from the midline pit nearest the anal verge by identifying subcutaneous strip-like hypoechoic areas. The connections between multiple pits were also identified. An appropriate incision was made at the sinus tract apex. If linear hyperechoic structures indicating broken hairs were observed, the hair and necrotic tissue were removed using mosquito forceps or a curette. Under continuous ultrasound monitoring, the laser fiber catheter was inserted through the pit and advanced to the sinus tract end. The sinus tract wall was ablated and closed by withdrawing the catheter at a constant speed of approximately 1 mm/s using a wavelength of 1470 nm and power of 10 W. Continuous real-time ultrasound visualization ensured complete ablation coverage of the entire tract and avoided damage to surrounding healthy tissue (Fig. 2).

To enhance the accuracy of ultrasound guidance and improve surgical outcomes in this study, radiology department ultrasound equipment was used during the procedure, and the surgery was performed with the assistance of a radiologist. All surgical components were performed by the same experienced colorectal surgeon.

### Postoperative management

Postoperatively, wounds were cleaned with povidone-iodine. Sterile gauze soaked with Kangfuxin solution was applied to the wound surface. Dressings were changed twice daily.

### Outcome measures and follow-up

(1) Treatment parameters, including laser duration and total laser energy used. (2) Occurrence of in-hospital postoperative complications (wound bleeding, pain). Postoperative pain was assessed using a visual analog scale (VAS) score (ranging from 0 for no pain to 10 for the worst possible pain). (3) Time to return to normal school and daily activities. (4) Sinus closure time. Sinus closure was defined as complete epithelialization of the incision. (5) Wound healing rate. Wound healing was defined as complete epithelialization of the wound surface without redness, swelling, or unusual secretions within 2 months post-surgery. If soiling persists beyond 2 months, the wound is considered unhealed. (6) Recurrence during the follow-up period. Recurrence was defined as the reappearance of swelling, pain, rupture, or purulent or serous discharge at the same site after complete wound healing. (7) Follow-up method: Postoperatively, patients were advised to keep the wound clean, avoid prolonged sitting and strenuous activities until fully healed, and participate in weekly online follow-ups by uploading wound photos and reporting any condition changes via an online platform until healing was complete.

### Statistical analysis

Statistical analysis was conducted using IBM SPSS 25.0. Normally distributed continuous variables were shown as mean ± standard deviation and compared with the independent samples* t* test. Non-normally distributed variables are presented as median (Q1, Q3) and compared using the Mann–Whitney *U* test. Categorical variables were presented as number (%) and were compared using the chi-square or Fisher’s exact test. Recurrence-free survival was assessed with the Kaplan–Meier method and log rank test. Multivariate logistic regression identified factors affecting recurrence rates. A two-sided *p* value of < 0.05 indicated statistical significance.

## Results

### Patient characteristics

The study included 64 patients, with 89.1% (*n* = 57) male and 10.9% (*n* = 7) female. Twenty-six patients underwent US-guided SiLaC, and 38 received SiLaC. The median age was 16.5 years in the ultrasound group and 17 years in the conventional group, with no significant age or sex differences (*p* > 0.05). The median follow-up was 18.0 months for the ultrasound group and 20.5 months for the conventional group, showing no significant difference (*p* = 0.875). Baseline characteristics were comparable (Table 1).

**Table 1:** Clinical characteristics of adolescent patients with sacrococcygeal pilonidal disease

Category	US-guided (*n* = 26)	Conventional (*n* = 38)	*p* value
Age	16.5 (15.0, 18.0)	17.0 (16.0, 17.5)	0.613
Sex			1.000
Male	23 (88.5%)	34 (89.5%)	
Female	3 (11.5%)	4 (10.5%)	
BMI	27.94 ± 4.36	26.91 ± 4.88	0.388
Duration (months)	4.5 (2.0, 6.0)	6.0 (3.0, 12.0)	0.057
Length (cm)	5.0 (3.8, 5.0)	5.0 (4.0, 5.0)	0.809
Diameter (mm)	9.0 (6.0, 10.3)	7.5 (7.0, 9.3)	0.529
Number of pits	2 (1, 3)	2 (1, 3)	0.580
Prior drainage			0.602
Yes	6 (23.1%)	11 (28.9%)	
No	20 (76.9%)	27 (71.1%)	
Prior pilonidal surgery			0.140
Yes	0	4 (10.5%)	
No	26 (100%)	34 (89.5%)	
Drinking history			0.561
Yes	2 (7.7%)	1 (2.6%)	
No	24 (92.3%)	37 (97.4%)	
Smoking history			0.784
Yes	1 (3.8%)	1 (2.6%)	
No	25 (96.2%)	37 (97.4%)	
Period			0.569
Inflammatory	10 (38.5%)	12 (31.6%)	
Stable	16 (62.5%)	26 (68.4%)	
Follow-up duration (months)	18.0 (8.0, 37.8)	20.5 (9.5, 35.8)	0.875

### Surgical efficacy and safety

**Fig. 3:**
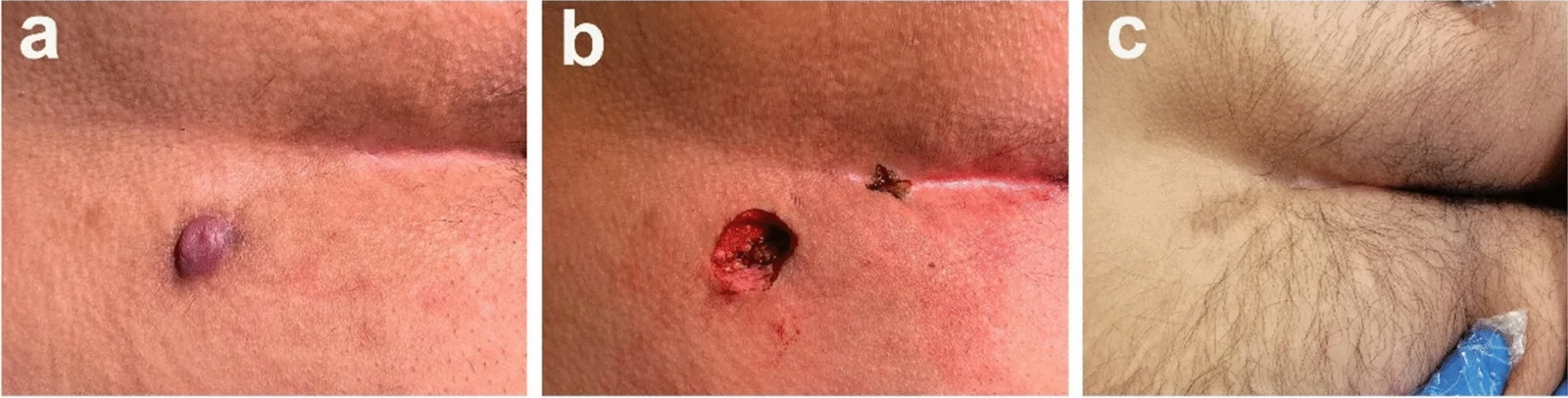
Sacrococcygeal photographs of a 16-year-old male patient with SPD who underwent US-guided SiLaC at different time points: **a** preoperative, **b** postoperative, and **c** 1 year after surgery

The ultrasound group had significantly shorter laser duration (41.1 s) and lower energy use (394.0 J) compared to the conventional group (54.1 s and 530.7 J, respectively), with *p* < 0.05. No patients in either group experienced intraoperative complications such as hypoxemia, hypotension, or bradycardia. Post-surgery, one ultrasound group patient reported pain, managed with oral analgesics, and resolved by the next day. Minor wound oozing occurred in one patient per group, controlled with pressure and topical agents. Median recovery to normal life was 11 days for the ultrasound group and 13 days for the conventional group, with no significant difference (*p* = 0.091). The ultrasound group had a significantly shorter median sinus closure time of 33.5 days compared to 39.5 days in the conventional group (*p* = 0.017). Healing rates were similar: 96.2% for the ultrasound group and 94.7% for the conventional group (*p* = 1.000). In the ultrasound group, the recurrence rate was 4% (1/25), while in the conventional group, it was 25% (9/36), with a significant difference (*p* = 0.037). This may suggest better outcomes for the ultrasound group (Table 2). Preoperative, postoperative, and 1-year postoperative images of a patient who underwent US-guided SiLaC are presented in Fig. 3.

**Table 2:** Surgical outcomes of adolescent patients with sacrococcygeal pilonidal disease

Category	US-guided (*n* = 26)	Conventional (*n* = 38)	*p* value
Laser time (s)	41.1 (18.8, 64.6)	54.1 (31.4, 86.6)	0.033
Energy (J)	394.0 (185.3, 640.3)	530.7 (306.2, 846.6)	0.036
Hospital stay (days)	4 (2, 6)	4 (3, 6)	0.331
Closure time (days)	33.5 (28.8, 36.5)	39.5 (31.0, 44.3)	0.017
Return to activity (days)	11.0 (9.8, 13.0)	13.0 (10.0, 14.0)	0.091
Complications, *n* (%)	2 (7.7%)	1 (2.6%)	0.561
Healing, *n* (%)	25 (96.2%)	36 (94.7%)	1.000
Recurrence/healing, (%)	1/25 (4%)^a^	9/36 (25%)^a^	0.037

^a^Percentage calculated on the basis of healed patients

A 15-year-old male patient in the ultrasound group achieved sinus closure in 66 days but still had minimal discharge, while two patients in the conventional group closed their sinuses in 42 days but experienced ongoing erythema and discharge after 2 months. These three patients were defined as non-healed, as postoperative symptoms persisted for more than 2 months. In the conventional group, nine patients recurred with a median time of 6.8 months; seven underwent repeat ultrasound-guided laser closure, and two had conventional laser closure. In the ultrasound group, one patient recurred at 5.2 months and received repeat US-guided laser closure. All 10 patients are currently being monitored with no further recurrences.

### Analysis of recurrence factors

A univariate analysis of 61 patients who healed post-surgery found no significant differences in sex, age, BMI, smoking and alcohol history, disease duration, sinus tract characteristics, disease stage, or previous surgeries between those with and without recurrence. However, recurrence was significantly associated with a higher rate of conventional surgery (*p* = 0.037) (Table 3).

**Table 3:** Univariate analysis of factors associated with recurrence

Category	Non-recurrence (*n* = 51)	Recurrence (*n* = 10)	Statistics	*p* value
Sex			*χ*^2^ = 0.000	1.000
Male, *n* (%)	46 (90.2%)	9 (90.0%)		
Female, *n* (%)	5 (9.8%)	1 (10.0%)		
Age	17.0 (15.0, 17.0)	16.5 (16.0, 18.0)	*Z* = − 0.481	0.631
BMI	27.19 ± 4.96	27.70 ± 3.45	*t* = 0.313	0.755
Smoking history			–^a^	1.000
Yes, *n* (%)	2 (3.9%)	0		
No, *n* (%)	49 (96.1%)	10 (100%)		
Drinking history			–	1.000
Yes, *n* (%)	3 (5.9%)	0		
No, *n* (%)	48 (94.1%)	10 (100%)		
Duration (months)	5.0 (3.0, 12.0)	6.0 (4.8, 12.0)	*Z* = − 0.453	0.729
Length (cm)	5.0 (4.0, 5.0)	4.5 (3.8, 5.0)	*Z* = − 0.619	0.549
Diameter (mm)	8.0 (6.0, 10.0)	8.5 (7.8, 10.3)	*Z* = − 1.331	0.187
Number of pits			*χ*^2^ = 0.160	0.738
≤ 1, *n* (%)	22 (43.1%)	5 (50.0%)		
> 1, *n* (%)	29 (56.9%)	5 (50.0%)		
Period			*χ*^2^ = 0.191	0.735
Inflammatory	19 (37.3%)	3 (30.0%)		
Stable	32 (62.7%)	7 (70.0%)		
Prior drainage			*χ*^2^ = 3.900	0.055
Yes, *n* (%)	15 (29.4%)	0		
No, *n* (%)	36 (70.6%)	10 (100%)		
Prior pilonidal surgery			–	0.521
Yes, *n* (%)	3 (5.9%)	1 (10.0%)		
No, *n* (%)	48 (94.1%)	9 (90.0%)		
Surgical procedure			*χ*^2^ = 4.546	0.037
Conventional	27 (52.9%)	9 (90.0%)		
US-guided	24 (47.1%)	1 (10.0%)		

^a^Fisher’s exact test was used when the expected frequency was less than 5

Variables with a *p* value less than 0.1 in the univariate analysis, in conjunction with prior surgical history and disease period identified as clinical risk factors in previous study [22, 26], were incorporated into a multivariate logistic regression model. Prior drainage was excluded from the model because of the absence of cases in the recurrence group, which resulted in complete separation and hindered the stable estimation of the regression coefficient. Following adjustments, the ultrasound group demonstrated a significantly reduced risk of recurrence compared to the conventional group (OR 0.091, 95% CI 0.020–0.406; *p* = 0.002). Notably, prior surgical history and disease period did not independently predict recurrence (*p* > 0.05) (refer to Table 4).

**Table 4:** Multivariable logistic regression analysis for recurrence

Category	β	SE	Wald χ^2^	*p*	OR	95% CI
Surgical procedure (US-guided vs. conventional)	− 2.397	0.763	9.859	0.002	0.091	0.020–0.406
Prior pilonidal surgery (Yes vs. no)	− 0.940	1.170	0.645	0.422	0.391	0.039–3.874
Period (Inflammatory vs. stable)	− 0.740	0.702	1.112	0.292	0.477	0.121–1.888

Model: Enter method, including core intervention factors with *p* < 0.1 and clinical risk factors. Prior drainage excluded due to complete separation. All VIF < 10, no collinearity. Hosmer–Lemeshow *p* > 0.05, good fit

### Comparison of recurrence-free survival

**Fig. 4:**
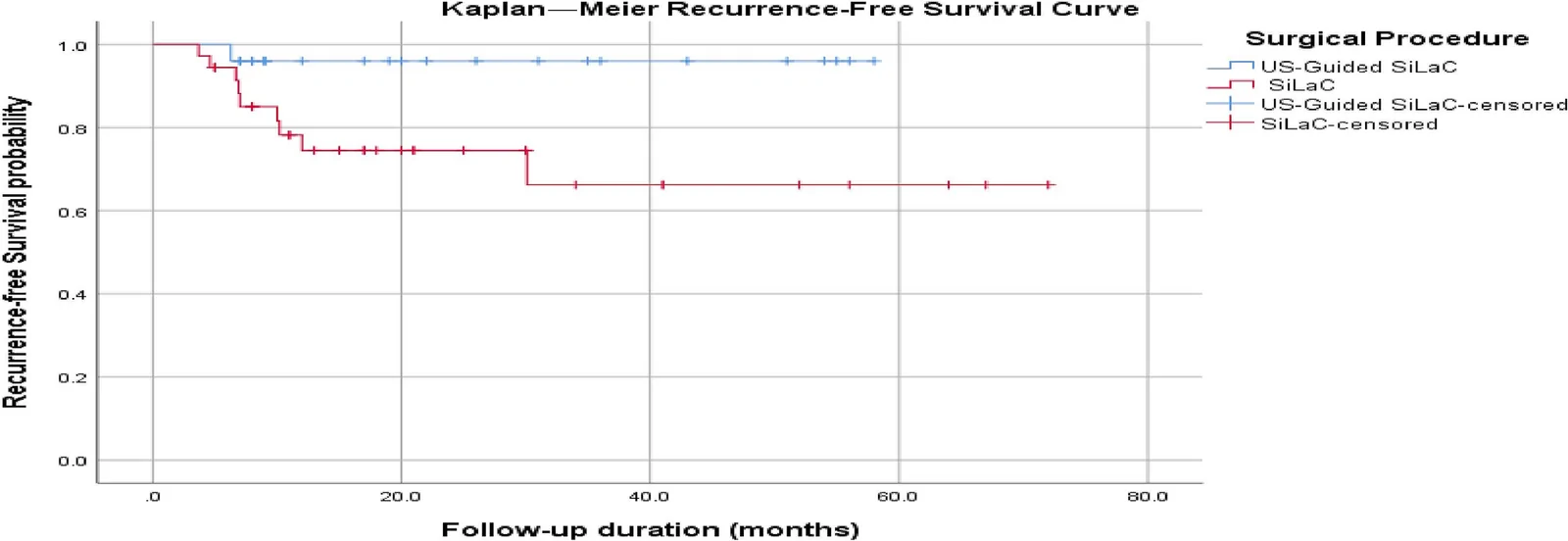
Kaplan–Meier recurrence-free survival curves for US-guided SiLaC and SiLaC groups. The US-guided SiLaC group showed a numerically higher recurrence-free probability, and the difference was statistically significant (log rank test, *p* = 0.034)

Kaplan–Meier survival analysis showed that the cumulative survival rate of the US-guided SiLaC group remained higher than that of the SiLaC group throughout the follow-up period (Fig. 4). The survival curve for the US-guided SiLaC group was relatively flat, maintaining a recurrence-free survival rate above 90% up to nearly 60 months of follow-up. In contrast, the curve for the SiLaC group showed a more pronounced decline between approximately 10 and 30 months of follow-up, eventually stabilizing at around 65%. The log rank test yielded a chi-square value of 4.507, with a *p* value of 0.034, suggesting a trend toward a survival benefit in the US-guided SiLaC group compared with the SiLaC group.

## Discussion

This retrospective cohort study represents the first systematic evaluation of the efficacy of ultrasound-guided SiLaC for SPD in the adolescent demographic. The findings suggest that, in this cohort, compared to conventional SiLaC technique, ultrasound-guided laser closure in adolescents with SPD was associated with intraoperative advantages and a lower recurrence rate during the short follow-up period. Multivariable logistic regression analysis indicated that the surgical procedure (US-guided vs. conventional) was a significant predictor of recurrence (OR 0.091, 95% CI 0.020–0.406; *p* = 0.002). Because the number of recurrence events was limited, the multivariable model should be interpreted cautiously and regarded as exploratory. Additionally, no significant differences were observed between the two groups concerning surgical success rate, safety, and functional recovery. Kaplan–Meier analysis revealed early and sustained recurrence-free survival benefits for the US-guided group. It appears to suggest that precise ultrasound-guided visualization might lower SPD recurrence post-SiLaC and enhance long-term disease control, but more prospective large-scale studies are necessary to validate these results and understand the mechanisms involved.

The fundamental advantage of utilizing ultrasound guidance lies in its capacity to provide real-time intraoperative visualization of the subcutaneous sinus tract structure [27]. While conventional laser ablation is considered minimally invasive, it is performed without direct visualization, relying on the surgeon’s tactile exploration and experiential judgment to estimate the course and branches of the sinus tract. Consequently, most intraoperative assessments do not accurately reflect the true anatomical boundaries, and may also create new iatrogenic sinus tracts as a result of improper use of the probe [25]. To overcome this limitation, the present study employed ultrasound guidance, which allowed the surgeon to clearly identify the primary sinus tract, its depth, extent, and any potential side branches not detected preoperatively or abscess cavities through imaging [28–30]. As a result, during the retraction of the laser fiber, energy could be precisely and comprehensively delivered to the entire sinus tract wall and its branches. This visualized operative approach facilitated more precise ablation, resulting in a reduced intraoperative laser time (median duration 41.1 s compared to 54.1 s) and decreased energy consumption (median energy 394.0 J compared to 530.7 J). The complete ablation of all sinus tract branches using laser energy under ultrasound guidance effectively eradicated residual diseased epithelial tissue, which is one of the anatomical bases for recurrence [31]. While precise ablation of sinus tracts is essential for reducing recurrence, other factors such as incomplete ablation, family history, high BMI, young age, sedentary lifestyle, excessive hair growth, and a deep natal cleft also contribute [7, 32–34]. The deep cleft can lead to new tracts by facilitating hair penetration and infection [35]. SiLaC removes existing tracts but does not change sacrococcygeal anatomy, so while it lowers recurrence rates (4% with ultrasound vs. 25% without), it does not prevent new tract formation. Flap-based procedures, however, such as the Bascom cleft lift, address anatomical issues by elevating the natal cleft and eliminating the unflattened crease; Ankersen et al. found that the recurrence rate of this procedure was only 19% in adolescents during a 5-year follow-up, an outcome that is certainly commendable [36].

Shorter laser duration and lower total laser energy resulted in better outcomes, demonstrating the precise targeted ablation achieved with real-time ultrasound guidance. This precise lesion targeting has transformed laser therapy for SPD from a high-energy, low-efficiency approach to a low-energy, high-efficiency one. Previous studies have demonstrated the high accuracy of ultrasound in assessing SPD [28, 29]; however, this study extends the application of ultrasound to the intraoperative phase, confirming its potential for real-time guidance and enhancing surgical precision. This finding is consistent with the perspective of Papagiannopoulos et al., who proposed that ultrasound-guided laser treatment delivers controlled energy exclusively to pathological tissue, thereby avoiding damage to healthy subcutaneous tissue [24, 29]. Minimizing unnecessary thermal injury helps maintain local microcirculation and promotes wound healing; this also explains the 6-day reduction in median sinus tract closure time in the US-guided SiLaC group compared with the SiLaC group. Ultrasound-guided laser ablation achieves a reasonable balance between the thorough elimination of diseased tissue and the preservation of healthy tissue, rendering this technique particularly valuable in the adolescent population.

Adolescents with SPD present distinct clinical characteristics that require individualized treatment approaches. Firstly, adolescence is the period with the highest incidence of SPD, marked by increased recurrence rates and earlier recurrence compared to adults [7, 37–39]. This phenomenon may be linked to hormonal changes that enhance pilosebaceous gland activity, genetic predisposition, the prolonged presence of the condition in adolescents, and more severe symptoms within this demographic [40–42]. Secondly, adolescents are in a critical phase of growth and academic development, with daily activities often involving high levels of physical activity or prolonged periods of sitting. These factors contribute to a lower tolerance for postoperative pain and poor adherence to long-term, frequent wound care, ultimately prolonging wound healing time. Furthermore, artificial intelligence (AI)-based predictions have corroborated that extended healing time is associated with an elevated risk of recurrence in pediatric SPD [43]. Consequently, an optimal surgical procedure for adolescent SPD should be radical, minimally invasive, and facilitate rapid recovery [44]. US-guided SiLaC does not constitute a fundamentally novel treatment modality; instead, it integrates intraoperative imaging to enhance the precision and comprehensiveness of the original procedure, thereby mitigating postoperative recurrence due to incomplete ablation. In this study, ultrasound-guided SiLaC exhibited superior outcomes in terms of sinus tract closure and short-term recurrence rates among adolescent patients. These findings suggest that ultrasound-guided SiLaC may help prevent early reoperation in this demographic, which is a notably promising result. However, it is important to acknowledge that the causes of SPD recurrence are multifaceted and complex. The observed benefits may represent a short-term advantage resulting from more thorough treatment of existing sinus tracts. Given that the anatomical and pathological basis of SPD remains unchanged, the long-term sustainability of the reduced recurrence rate achieved through ultrasound guidance remains uncertain.

In addition to its direct clinical advantages, the utilization of ultrasound guidance in pilonidal sinus surgery offers substantial potential for standardization. Traditional SiLaC techniques predominantly rely on the surgeon’s expertise and tactile spatial perception, which can result in significant variability in treatment outcomes. The incorporation of ultrasound technology transforms this procedure into a visualized surgery, thereby diminishing reliance on subjective tactile feedback. This objectification of the surgical field is anticipated to reduce the learning curve for practitioners and promote the broader adoption of the technique. Future advancements may include the integration of AI algorithms to aid in the real-time interpretation of ultrasound images, thereby enhancing the ability to identify complex sinus tract branches and paving the way for intelligent, automated ablation systems.

Despite the contributions of this study, several limitations must be acknowledged. Firstly, the sample size, comprising 64 patients, is relatively small, and the single-center retrospective design may introduce selection bias. Therefore, the observed difference in treatment outcomes, although statistically significant, may only be hypothesis-generating: the difference arises from the intraoperative technical advantage of ultrasound guidance. Secondly, the median follow-up period of approximately 19 months, while sufficient for assessing short- to medium-term recurrence, requires extended observation to evaluate long-term efficacy comprehensively. Thirdly, the evaluation of healing and recurrence was based on clinical judgment, lacking objective imaging confirmation. Future studies could benefit from incorporating ultrasound during follow-up to assess wound status more accurately. Furthermore, the limited sample size precluded comprehensive subgroup analyses. The potential advantages of ultrasound guidance in identifying occult lesions and mitigating interference from scar tissue in complex cases, such as those with prior surgical history or multiple pits, remain unexplored and merit further investigation. Prospective, multicenter, large-sample randomized controlled trials are essential to validate the long-term efficacy and explore the utility of this technique in adolescent patients with complex sinus tracts or recurrent SPD.

## Conclusions

These preliminary results indicate that ultrasound-guided laser ablation may represent a safe and effective minimally invasive treatment modality for selected adolescent patients with SPD, particularly for patients who desire shorter postoperative wound recovery time and reduced risk of recurrence. By substituting the blind procedure in SiLaC with a vision-guided approach, this technique offers novel avenues for advancing precision and personalized treatment of adolescent SPD in the future.

## Data Availability

No datasets were generated or analysed during the current study.
